# Experience of participating in a randomised controlled feasibility trial of surgery versus no surgery as part of multimodality treatment in stage III-N2 non-small cell lung cancer: PIONEER

**DOI:** 10.1136/bmjresp-2026-004632

**Published:** 2026-10-26

**Authors:** Melissa Betty Perry, Ashleigh Ward, David R Baldwin, Fiona Blackhall, Charlotte Eastwood, John Edwards, Matthew Evison, Corinne Faivre-Finn, Seamus Grundy, Emily Harris, Neal Navani, Sarah Rhodes, Jonathan Robson, Selina Tsim, Paul Van Schil, Ian Woolhouse, Janelle Yorke, Sally Taylor

**Affiliations:** 1 Christie Patient Centred Research, The Christie Hospital NHS Trust, Manchester, UK; 2 Respiratory Medicine, Nottingham University Hospitals NHS Trust, Nottingham, UK; 3 University of Nottingham, Nottingham, UK; 4 The Christie NHS Foundation Trust, Manchester, UK; 5 Manchester Biomedical Research Centre, The University of Manchester, Manchester, UK; 6 Department of Cardiothoracic Surgery, Sheffield Teaching Hospitals NHS Foundation Trust, Sheffield, UK; 7 Lung Cancer and Thoracic Surgery Directorate, Wythenshawe Hospital, Manchester, UK; 8 Manchester Academic Health Science Centre, The University of Manchester Faculty of Biology Medicine and Health, Manchester, UK; 9 Northern Care Alliance NHS Foundation Trust, Manchester, UK; 10 Lungs for Living Research Centre, UCL Respiratory, University College London, London, UK; 11 Department of Thoracic Medicine, University College London Hospitals NHS Foundation Trust, London, UK; 12 Centre for Biostatistics, The University of Manchester, Manchester, UK; 13 Department of Cardio-Respiratory, Leeds Teaching Hospitals NHS Trust, Leeds, UK; 14 Respiratory Medicine, Queen Elizabeth University Hospital, Glasgow, UK; 15 School of Cancer Sciences, University of Glasgow, Glasgow, UK; 16 Department of Thoracic and Vascular Surgery, University Hospital Antwerp, Edegem, Belgium; 17 Respiratory, University Hospitals Birmingham NHS Foundation Trust, Birmingham, UK; 18 Queen Elizabeth Hospital, Birmingham, UK; 19 School of Nursing, The Hong Kong Polytechnic University, Hong Kong, Hong Kong; 20 Division of Nursing, Midwifery and Social Work, The University of Manchester Faculty of Biology Medicine and Health, Manchester, UK

**Keywords:** Non-Small Cell Lung Cancer, Surveys and Questionnaires, Psychology

## Abstract

**Introduction:**

Multimodality treatment is recommended for potentially resectable stage III-N2 non-small cell lung cancer. However, no clear evidence states that surgery benefits survival and health-related quality of life (HRQoL). This multicentre (n=10, but 5 actively recruited) study explored experiences of patients, carers and multidisciplinary teams (MDTs) participating in a feasibility trial to inform the design of a phase III randomised trial with HRQoL as a primary outcome.

**Methods:**

Patient participants were randomised to receive multimodality treatment with or without surgery. Recruitment consultations were audio-recorded and analysed using content analysis. Patients and their informal carers completed weekly diaries. Semistructured interviews were conducted with declining and consenting patients, consented carers and MDT members. Interviews and diary data were analysed using framework analysis.

**Results:**

28 patients and 9 carers participated in the PIONEER trial. 12 patients, 2 carers, 4 consent-declined patients and 20 clinicians were interviewed individually. Recruitment consultations of 4 declining and 12 consenting patients were recorded. 21 patients and 8 carers completed diary entries. All data sources were combined during analysis to create three overarching concepts: (1) Trial processes (treatment explanation, decline reasons, information needs, recruitment and eligibility, MDT engagement, patient factors and system issues); (2) Experience of PIONEER participation (daily impact physically and psychologically and coping mechanisms used) and (3) Considerations for a future trial (design, protocol, disease definitions, recruitment and engagement).

**Conclusions:**

Patient and carer experience of participating in PIONEER was generally positive, though trial design improvements such as electronic questionnaires and diaries were recommended. Clinicians highlighted concerns regarding treatment equipoise, resectability assessment and the limited pool of eligible patients. These findings help explain the recruitment difficulties experienced and suggest that future research may be better directed towards pragmatic real-world study designs and improving treatment access for the large proportion of patients currently deemed ineligible.

**WHAT IS ALREADY KNOWN ON THIS TOPIC**

- Although survival rates after multimodality treatment for stage III-N2 non-small cell lung cancer (NSCLC) are similar whether or not surgery is included, little is known about the impact on patients’ and carers’ health-related quality of life (HRQoL), symptoms, treatment burden and psychological well-being. Recruitment to randomised controlled trials comparing different treatment modalities has historically been limited and challenging.

**WHAT THIS STUDY ADDS**

- First evaluation of the feasibility of randomising this patient group to surgery versus no surgery, with HRQoL as the primary outcome captured through weekly diaries and in-depth interviews.

**HOW THIS STUDY MIGHT AFFECT RESEARCH, PRACTICE OR POLICY**

- By combining quantitative and qualitative evidence, this study demonstrates that recruitment barriers in stage III-N2 NSCLC are largely structural rather than trial-specific. The findings support a shift towards pragmatic real-world and adaptive study designs, greater attention to patients excluded from current treatment pathways and continued integration of HRQoL into treatment decision-making to support patient-centred care.

## Introduction

 In the UK, approximately 80%–85% of lung cancers are classified as non-small cell lung cancers (NSCLC).^1^ Stage III-N2 is characterised by nodal metastases to the mediastinum on the same side as the primary tumour, representing approximately 6% of all lung cancer cases.^2^ Symptoms, including chest pain, chronic cough, shortness of breath, weight loss and tiredness, can impact health-related quality of life (HRQoL).^3^ Research suggests patients often characterise HRQoL into four domains that influence their treatment decisions: somatic, functional, relational and emotional.^4^

Stage III-N2 NSCLC curative-intent treatment is multimodality with systemic anti-cancer therapy (SACT), to prevent metastatic disease, and with surgery, radiotherapy or both for local primary tumour and nodal control. Current evidence fails to exhibit the distinct superiority of regimens including surgery over others.^5–8^ Intensive treatment regimens may also cause significant impacts on patients’ and carers’ HRQoL. There are likely to be noticeable differences in patient experiences, and the treatment side effects and burden between these two treatment pathways; however, stage III-N2 NSCLC-related literature focusing on survival outcomes is severely lacking in these person-centred metrics.^9^ Such data are essential to support patients, carers and clinicians to make an informed treatment decision. The limited research, specifically on carers for lung cancer patients, highlights the high caregiving burden, reduced HRQoL and economic impact that caregiving for someone with lung cancer can have.^10–12^ However, no research has explored whether this carer experience differs depending on the patient’s treatment pathway, which could ultimately help support patients in shared decision-making.

The evidence to support cancer treatment options and care is, in some cases, complex, limited and incomplete, where information on the risks and benefits of each treatment is often unwanted or interpreted differently among patients.^13 14^ These obstacles can make recruitment to randomised controlled trials (RCTs) challenging, due to methods of identifying eligible patients and conveying the equipoise of treatments during consultations.^15^ Our PIONEER trial^16 17^ aimed to assess the feasibility of recruiting and randomising patients with ‘potentially resectable’ stage III-N2 NSCLC and their carers to either surgery or no surgery. Collecting person-centred data, such as comparative HRQoL data and the burden of multimodality treatment, will support a more holistic approach to cancer care, informing both treatment decisions, supportive needs and the design of future studies. Therefore, within PIONEER, we explored the experiences of patients, carers and multidisciplinary teams (MDTs) in relation to the recruitment process, trial participation, treatment burden and considerations for a future fully powered trial with HRQoL as a primary endpoint.

## Methods

### Study design

A UK-based multicentre feasibility RCT where patients with stage III-N2 NSCLC were randomised to either a surgical arm (with or without radiotherapy, plus SACT) or a non-surgical arm (radiotherapy and SACT). The protocol and quantitative elements of the main study have been published.^16 17^ This article focuses on the qualitative elements of the main study and adheres to the Consolidated Criteria for Reporting Qualitative Research (COREQ) guidelines (online supplemental file 1).^18^ The QuinteT Recruitment Intervention (QRI)^19^ was used as a method to identify and address recruitment issues. The methodology includes five stages: (1) Understanding the recruitment process; (2) Documenting and understanding barriers to recruitment; (3) Assessing how the trial processes and treatment regimens are being delivered by recruiters; (4) Presenting evidence and strategies to overcome recruitment difficulties to the trial management group and (5) Implementing the plan of improvement.^19^ One of the key elements of the QRI applied was recording recruitment consultations. Real-time feedback of consultations was presented in trial management group meetings (attended by a representative from each site) in order to rectify this barrier to recruitment and emphasise the importance of treatment equipoise. Summary information sheets including examples of good and poor practice were also developed and circulated with recruiting sites. Additionally, as part of the QRI process, anonymised data were collected for all patients assessed for PIONEER eligibility (data not presented in this paper), resulting in management meetings with individual sites and modifications to eligibility criteria.

#### Patient inclusion and exclusion criteria

Inclusion criteria for the whole PIONEER trial were as follows: being aged over 18, having potentially resectable T1–4 N2 M0 NSCLC, receiving an MDT recommendation for multimodality treatment, an MDT consensus that the patient has adequate physiological reserve for multimodality treatment, that either treatment arm is both technically and clinically appropriate, and being able to provide informed consent.

#### Sample size

We planned to interview approximately 12–16 patients and carers, 10 declining patients and 20–30 MDT members, with recruitment stopping when data saturation was reached.

#### Patient and public involvement

The study was presented at the Wythenshawe/Christie lung cancer patient and public involvement group and the North Trent Consumer Research Panel. Members were asked to provide feedback on the study and the methodology used. Many attendees had concerns about treatment being randomised and gave suggestions as to how this should be explained to patients in the documentation and during the recruitment process. A patient co-applicant reviewed the study protocol to ensure that the real-life experiences of patients were considered throughout and reviewed all patient-facing documents to ensure they were clear and understandable. The PPI co-applicant also attended trial management meetings. Following publication, participants will be provided a plain English summary of the study results.

### Data collection

#### Recruitment and consent

From September 2020 to April 2023, patients with stage III-N2 NSCLC were identified during MDT meetings across 10 UK sites. Patients were approached in clinic by the clinical team, usually a respiratory physician. Participant information sheets were provided, with the opportunity to ask questions. Patients gave written informed consent, including consent to all qualitative elements of the study. Patients were invited to nominate a carer (family member or friend) to participate and provide written informed consent.

#### Procedures

Interviews were conducted by four female research assistants (MBP, AW, CE and EH), who had experience in conducting qualitative interviews with oncology patients. The researchers were academics with no clinical background in lung cancer or preconceived biases towards treatment. Participants had not met the interviewer before but were aware that they worked in a research team at a cancer centre and were not part of their clinical care. No field notes were taken, transcripts and findings were not returned to participants for input or corrections, and no repeat interviews were carried out. As interviews were conducted over the phone, we cannot verify whether non-participants were present. Information on the different methods of data sources collected, time points, consent, transcription, methods and reason for collection can be found in table 1.

**Table 1:** Details of data sources collected, and the methods and processes following the QuinteT Recruitment Intervention methodology

Data source	Time point	Consent	Methods of data collection	Reason for collection
Recruitment consultations (n=16)	During the recruitment consultation with a member of the clinical team.	Verbal consent, written consent retrospectively.	All recruiting sites were sent a password-protected Dictaphone. Face-to-face consultations between the potential participant and member/s of the clinical research team were audio-recorded and detailed notes were created as per analysis. Consultations were 27 min long on average.	To optimise recruitment, identify any recruitment problems that may stem from the way the study was being presented to patients during the initial consultation and provide real-time feedback to clinicians to address any barriers to recruitment
Interviews with patients who declined to take part (n=4)	Within 1 week of a potential participant indicating that they did not wish to take part.	Consent was taken by the clinical team at the time of decline.	A semistructured interview guide was developed by the research team and patient and public involvement contributors. Due to geographical location, interviews were conducted and audio-recorded via telephone and transcribed verbatim. Interviews were 8 min long on average.	Reasons for decline, feedback on the randomisation process and treatment options and any suggested changes that would improve participation.
Weekly diary entries completed by patients (n=21 at least one time point; n=4 all time points) and their carers (n=8 at least one time point; n=1 all time points).	Weekly for the 24-week duration of the study. Diaries were collected at four time points: weeks 6, 9, 12 and month 6	Consent was taken as part of the informed consent form.	Five questions were developed by the research team and patient and public involvement contributors. Respondents rated their experience on a scale of 1–5, and for each question, there was also space to give a free-text response. Participants completed paper diaries in the clinic or at home.	How their daily activities and mood had been impacted by their illness, treatment or their caring responsibilities, how they felt compared with the week before, and if the number of hospital appointments affected their daily life.
Interviews with participating patients (n=12) and carers (n=2)	On completion of all their study procedures, scheduled at their convenience.	Consent was taken as part of the informed consent form.	A qualitative semistructured interview. An interview schedule was developed by the research team and reviewed by patient and public involvement contributors. Due to geographical location, interviews were conducted and audio-recorded via telephone and transcribed verbatim. Interviews were 20 min long on average for patients and 6 min for carers.	To discuss their experience of treatment, trial participation and to determine if there were any changes required for future trials.
Interviews with members of the multidisciplinary team (n=20)	On recruitment completion across all sites.	Consent was taken as part of the informed consent form.	Purposive sampling was based on the level of patients deemed eligible at each site when screening (no eligible patients; low ineligibility (1%–59% of patients screened were ineligible); average ineligibility (60%–80% of patients screened were ineligible); high ineligibility (above 80% of patients screened were ineligible)) and professional grouping (surgeon, respiratory physician, oncologist, other). Due to geographical location, interviews were conducted and audio-recorded via telephone or Microsoft Teams and transcribed verbatim. Interviews were 16 min long on average.	To ascertain barriers and facilitators to recruitment and trial processes.

### Data analysis

Qualitative data generated from interviews and diaries were analysed using framework analysis by three female researchers (MBP, AW and EH).^20^ Framework analysis is useful to inform future practice and research design rather than theory development^21^ and allows researchers to adopt inductive and deductive approaches,^22^ therefore, answering specific questions relating to participation barriers and facilitators, but also allowing inductive themes relating to experience to emerge. Key steps of framework analysis include familiarisation, development, application and charting, applied to each data source independently using NVivo. ST acted as a mediator for coding disagreements.

Quantitative diary data were explored descriptively to identify patterns of experiences over time and differences between treatment arms. Audio recordings of recruitment consultations were analysed using content analysis^23^ to highlight specific issues and enable real-time feedback to the trial management group and to clinicians. Key themes were recorded and included in subsequent analysis.

Analysing each data source individually highlighted the distinct viewpoints and perspectives of participants. At the final stage of interpretation, the analysis of each data source, including content analysis of consultations, was combined to create overarching concepts.

## Results

### Participants

28 patients and 9 carers participated in the main trial. 16 patients agreed to record their recruitment consultations; four of these declined to participate in the main trial. 12 patients, 2 carers, 4 patients who declined trial participation and 20 MDT members participated in interviews. Details of interview participants and interview length are included in table 1. The patients and carers interviewed represented four of the five MDT sites that recruited participants and recruitment consultations were received from three sites. Each site was represented in the MDT interviews. 16 patients, 7 carers and 11 MDT members were not interviewed. Participant characteristics are shown in online supplemental table 2; no demographics were collected for decliners. To enhance theme reporting, quotes are presented in online supplemental table 3.

### Overarching framework

Three overarching concepts were developed from combined data analysis: (1) trial processes; (2) experience of taking part in PIONEER and (3) considerations for a future trial (figure 1).

**Figure 1:**
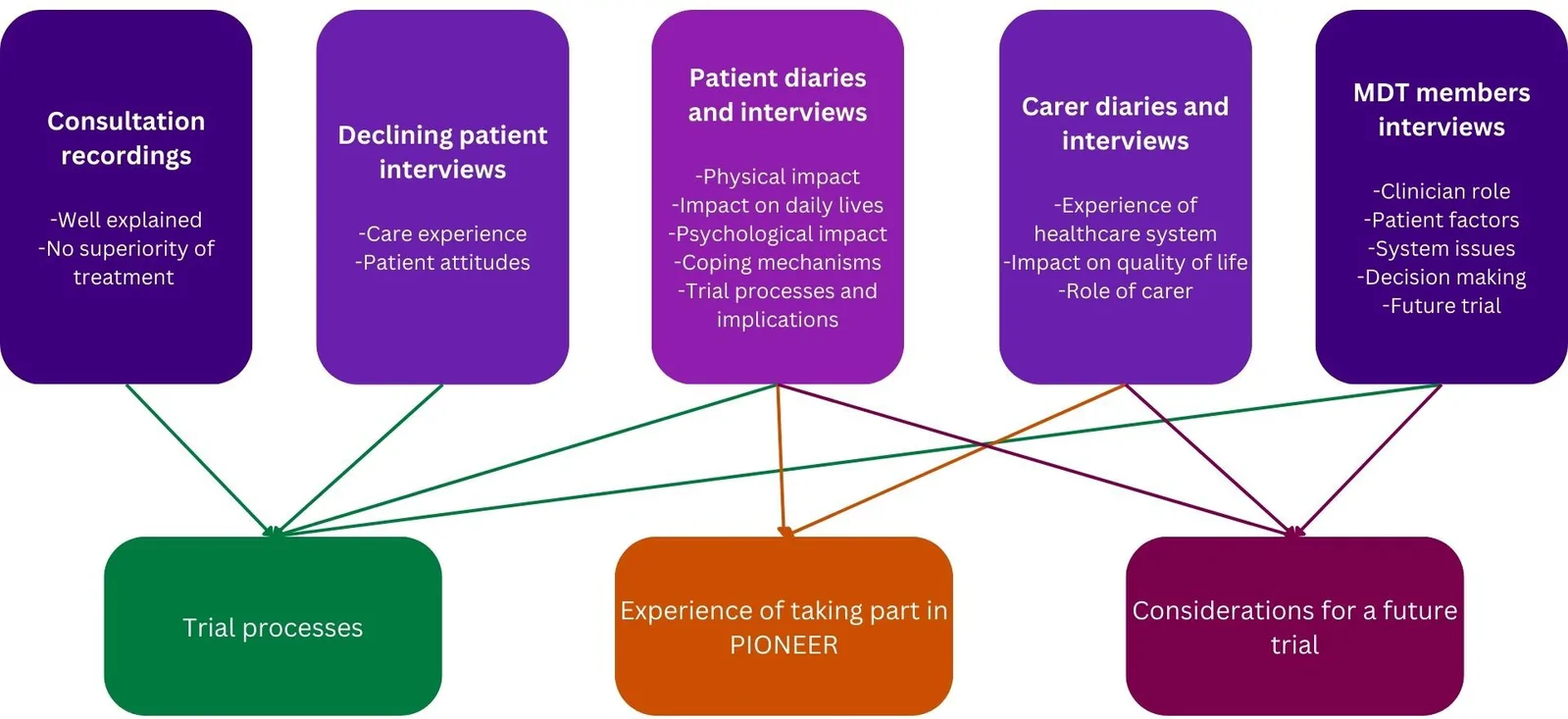
Framework analysis. MDT, multidisciplinary team.

### Trial processes

Key themes related to trial processes within the recruitment consultations and interviews with consenting and declining patients included: information delivery; randomisation concerns and decision-making.

#### Information delivery

Generally, PIONEER was explained well; clinicians emphasised the lack of evidence demonstrating treatment superiority. In most cases, clinicians gave equal weighting when explaining treatments and discussing logistics and potential side effects. Many consenting and declining patients reported feeling satisfied with the information and understood the trial and treatment information. Patients particularly valued the written information received. Prior cancer experience and treatment made some patients feel better prepared and more informed about treatment options.

#### Randomisation concerns

The majority of patients were not concerned about randomisation, but a few seemed unsure about the logistics of the randomisation process. One patient misunderstood randomisation and assumed they could choose their own treatment. For one patient, it was the randomisation element of the trial which made them decline participation; they wanted to have control over their treatment decision rather than it being made at random.

#### Decision-making

Patients used the information provided by the clinical team in their decision-making but also spoke to family and friends. The way the information was delivered by clinicians was crucial in decision-making. Occasionally, more attention was given to one treatment over another; for example, surgery side effects were heavily discussed but limited for radiotherapy. Consequently, patients decided against surgery and declined participation. Two patients preferred surgery, thinking it meant faster treatment (completed in 1 day), whereas radiotherapy included several visits over many weeks. The physical impact of the operation and previous experience were key drivers of preference against surgery. Altruism was the most common motivation for trial participation.

#### Interviews with MDT members

The focus of interviews with the MDT members was recruitment, with key themes identified as: clinician role, patient factors, system issues and decision-making.

#### Clinician role

Clinician involvement was critical to successful PIONEER recruitment. Clinicians actively attended MDTs and championed PIONEER to avoid it being overlooked. The more PIONEER was mentioned, the more engagement increased. It was felt that not all MDT members were aware of PIONEER or fully understood the complexities. Recruitment was difficult when MDT members had a bias towards their specialty or other trials. These created challenges when opinions regarding treatment equipoise differed, for example, believing surgery should always be offered where feasible. Part of the eligibility criteria included no strong preference from clinicians that there was a superior treatment option. As a result, any opinions about lack of treatment equipoise or bias towards treatment made it difficult for clinicians to identify eligible patients. The PIONEER trial was complex; therefore, it was important to allow sufficient time for the consultation. Clinicians needed to listen to patient cues and tailor conversations accordingly. Some clinicians emphasised the importance of informing patients that they could still withdraw from the trial if dissatisfied with the randomisation outcome. Another stressed the importance of mentioning additional monitoring that completing trial HRQoL questionnaires would bring.

#### Patient factors

Some clinicians felt patients would decline trial participation due to treatment randomisation or having a treatment preference. They discussed patient preconceptions, such as better success with surgery due to tumour removal, or concerns about having an operation. Clinicians stated the importance of addressing preconceptions and presenting an unbiased view of the treatment options. Some clinicians felt patients may decline due to reluctance to complete study questionnaires. Clinicians also highlighted concerns over the limited pool of eligible patients, whereas others expected a higher eligibility rate.

#### System issues

Healthcare system issues impacted identification of eligible patients and recruitment in PIONEER. There were often multiple trials patients could be eligible for; therefore, PIONEER could be overlooked or patients allocated to other trials. Several clinicians felt the PIONEER referral and recruitment pathway was more complex than many other trials. Patients were not always recruited through their referring centre, requiring an extra visit to a different site for study discussion. Clinicians highlighted the lack of resources, including limited support from research teams due to staffing levels or limited time from the clinicians due to competing commitments, may have had an impact on identifying suitable participants.

#### Decision-making

Patient referral involved a detailed MDT discussion, considering physiological fitness and resectability. MDT members mentioned specific steps within their decision-making processes. First, determining if the patient was eligible for surgery, if confirmed, the MDT discussed PIONEER suitability and explored any strong views from the clinical team regarding treatment. Various MDT members felt that all stage III-N2 patients should be offered PIONEER with less emphasis on an MDT decision. Some surgeons felt surgery should be offered to all potential surgical patients and therefore did not recommend PIONEER. One MDT member mentioned that surgery was their default position, assuming the patient was operable, and then looking at reasons why not. Main reasons for ineligibility were lack of physiological fitness, unresectable disease or both.

### Experience of taking part in PIONEER

#### Interviews with consenting patients and their carers

Four key themes from patient and carer qualitative interviews and diary data emerged: physical impact, impact on daily lives, psychological impact and coping mechanisms.

##### Physical impact

Surgical patients generally described more physical problems than patients treated with radiotherapy. Radiotherapy side effects reported were minimal, but patients discussed various chemotherapy side effects. They spoke about deterioration and improvements in their health and the importance of allowing time to rest to help manage symptoms and aid recovery. Generally, they felt their symptoms and side effects were well-managed by the clinical team. Carers spoke about the physical impact of the treatment and recovery on the patient, but also the impact their caring role had on their own physical health. They felt tired or exhausted due to the demanding treatment and appointment schedule and sleep was often affected.

##### Impact on daily lives

For radiotherapy patients, appointments were frequent, significantly impacting patients’ and carers’ daily lives. Long days at the hospital were made even longer if travelling a distance or using hospital transport. Patients tried maintaining daily activities but often made modifications such as doing them at a slower pace. Carers tried to maintain normality but sometimes had to adapt their routine. Some carers described rearranging or cancelling social plans due to caring responsibilities. Carers were generally happy to support their relatives, but the role often took its toll on them. Many carers had other demands, such as childcare and work commitments and juggling this with caring responsibilities was a challenge. One patient mentioned the financial impact due to their spouse changing their working hours. Over time, patients felt they could slowly start to do more and were gradually getting back to normal and regaining independence. Carers also commented on life slowly getting back to normal following treatment.

##### Psychological impact

Feelings of anxiety were common among patients, related to aspects of treatment or future worries. Many patients described feeling low, depressed or lonely. Frustrated by their lack of independence, patients felt a burden to others. Socialising was limited but contact with friends and family gave patients a break or something to look forward to. Patients’ moods fluctuated, experiencing highs and lows, improving as patients moved through treatment or physical health improved. Patients tried to maintain a positive outlook; they saw treatment as something they just had to endure, and felt their strength and resilience helped through this difficult time. Only one patient mentioned access to psychological support via a support group. Patients felt relieved when completing treatment or, in some cases, starting, knowing things were moving forward. Hospital visits, delays or poor communication often caused feelings of stress.

Carers reported worry and anxiety relating to different elements of patient care. Some reported treatment concerns, particularly surgery, while others were concerned about deteriorating health or the future. They felt frustrated seeing loved ones unwell, unable to help them. Carers reported psychological strain for both them and the patient, having little time for themselves. Some carers mentioned trying to keep their emotions hidden to avoid upsetting the patient. Carers also reported feeling down on several occasions, sometimes reflective of how the patient was feeling. For some carers, their mood began to improve as the patient moved further into recovery, feeling relieved when they could see this improvement. Some carers tried to maintain a positive mindset.

##### Coping mechanisms

The support patients received was crucial in helping them cope with treatment. The patients’ carer was a key part of their support network, and they appreciated this. Carers were happy to fulfil their role and did not see it as a burden. Their role was multifaceted, including taking the patient to appointments, helping with tasks at home during treatment, providing emotional support and advocating for the patient when required. In addition, patients often had a wider support network, something which carers found invaluable.

Additionally, clinical team support and communication helped, with patients and carers generally satisfied with the support and information they received. However, a few patients reported problems with communication, such as not having enough information about the different stages of their treatment, difficulties contacting people on the phone or feeling like they were not given enough notice to prepare for surgery. Most patients and carers felt well supported and knew they could access additional support when required. One patient, however, did mention a lack of support and felt that support reduced over time.

### Quantitative responses from diaries

#### Patients

Figure 2 presents responses to the quantitative diary questions; the 1–5 scale is colour coded with dark green representing the most positive experience and red representing the most negative experience. In the surgical arm, daily activities seemed to be more heavily impacted between weeks 6 and 16, whereas in the non-surgical arm, the first 8 weeks. Impact on mood varied; some reported no impact at all, whereas others reported they were ‘very much’ impacted. Others reported their mood as changeable from week to week. There were mixed responses from patients regarding how they felt compared with the previous week. Patients in the non-surgical arm seemed much more impacted by the number of appointments, particularly during the first 12 weeks of the study.

**Figure 2:**
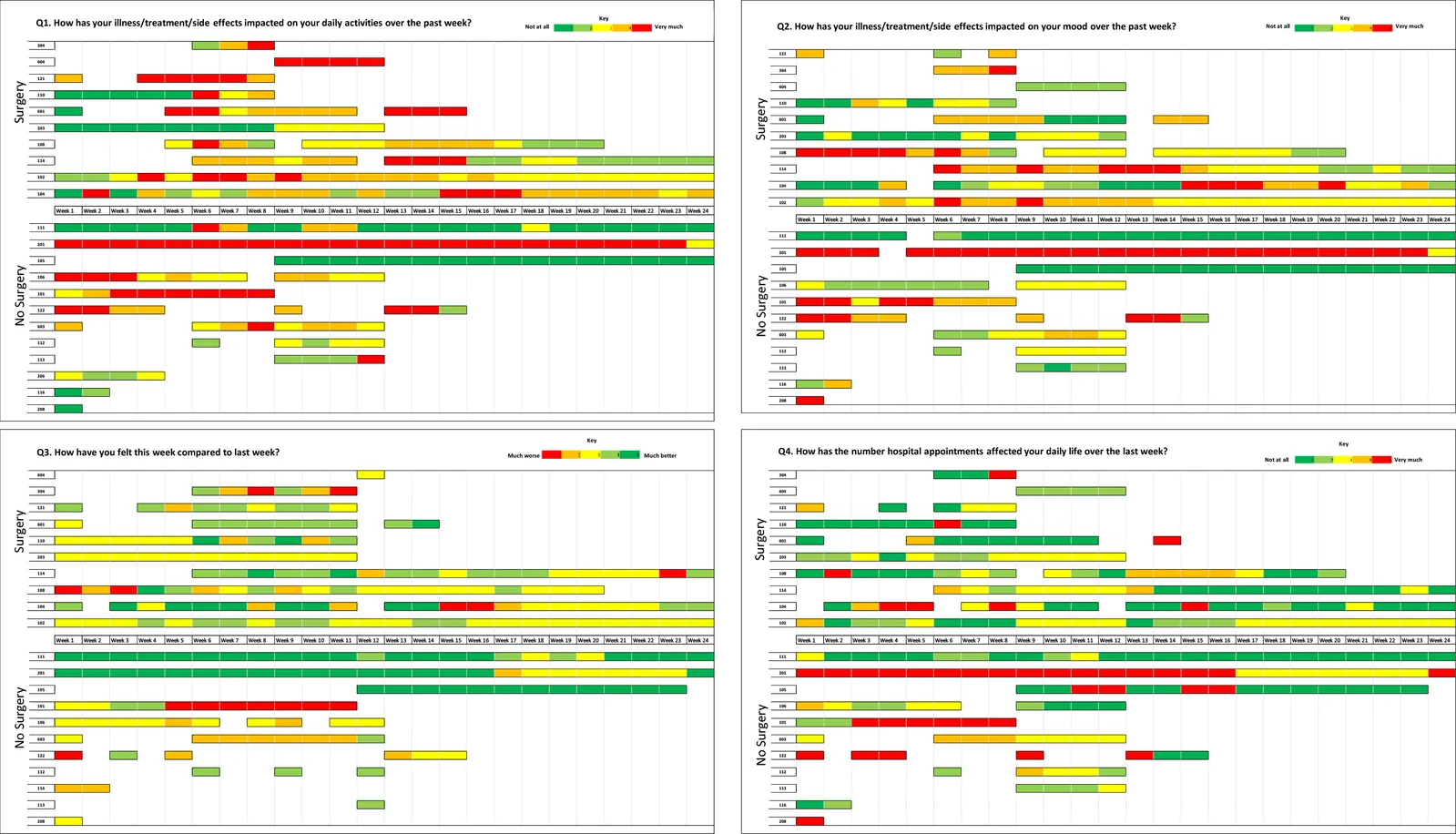
Patient quantitative diary entries.

#### Carers

One carer (non-surgical arm) completed diary entries from weeks 1–12 and reported their daily activities had been ‘very much’ impacted at every time point, as presented in figure 3, whereas most other carers reported little impact. In general, carers’ mood seemed to be impacted more heavily between weeks 1 and 4. There were also mixed responses from carers regarding how they felt compared with the previous week. Any instances where carers said they felt ‘much worse’ were all reported between weeks 2 and 6. Most carers did not feel they had been severely impacted by the number of hospital appointments. Only one carer (surgical arm) stated they had been ‘very much’ impacted at just one time point (week 8); two carers (non-surgical arm) said their lives had been affected ‘quite a bit’ at week 1.

**Figure 3:**
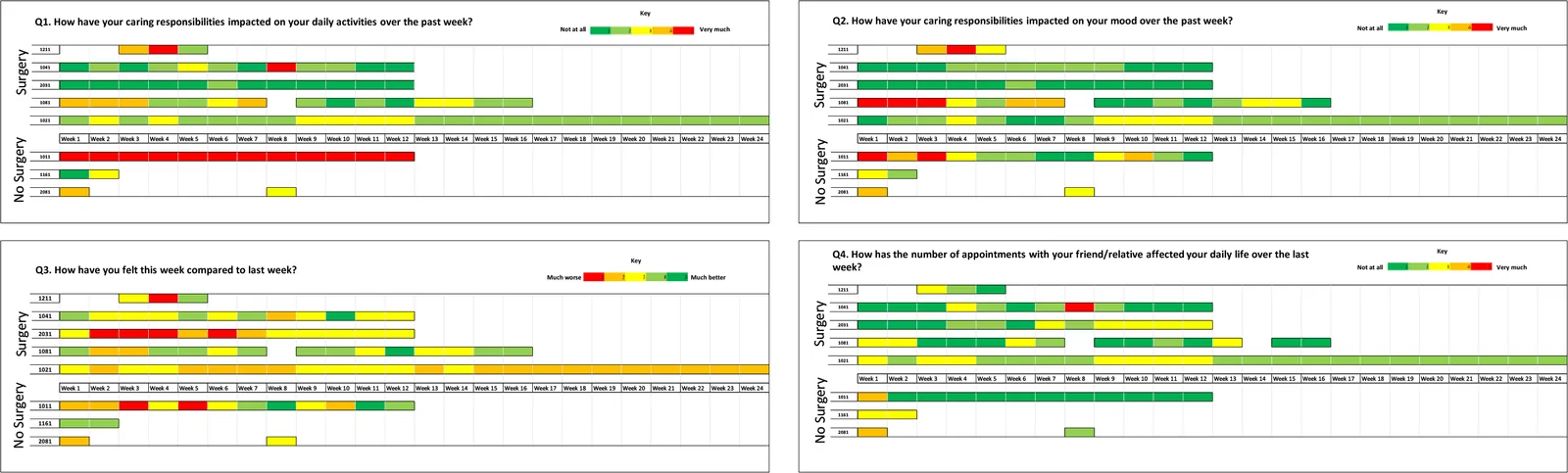
Carer quantitative diary entries.

### Considerations for a future trial

#### Interviews with consenting patients and their carers

The main comments from patients and carers regarding trial design improvements related to the questionnaires and diaries, commenting on the number and difficulty completing them all and on time. Patients often found the questionnaire and diary dates confusing or content repetitive, particularly if they did not feel much had changed since their last completion. Participants found the diaries more time-consuming due to the free text responses and one carer mentioned finding it difficult to find the time to complete them among their other commitments. Some patients would have preferred to complete questionnaires and diaries online.

#### Interviews with MDT members

Clinicians made several recommendations for future trials:

- Flexible protocol that can be adapted in line with new treatments and updated clinical guidance.
- Broader eligibility criteria to improve recruitment.
- Greater clarity around equipoise between treatments and the definition of resectable disease.
- Appointment of a dedicated MDT member to champion the study.
- Increased awareness and engagement, particularly from surgeons, supported by structured processes (eg, information sessions, checklists to make sure the trial is fully explained and more accessible trial information in MDTs).
- Introduction of a second eligibility screen following MDT discussion.
- Enhanced collaboration between sites to explore and address differences in recruitment rates.
- Reduction in the number of questionnaires for participants.
- Presentation of the study to patients by an MDT with representatives from each professional group.

Further discussion of these considerations can be found in the main study paper.^17^

## Discussion

This paper provides novel insights into the impact of different treatment pathways, surgery versus no surgery, on the HRQoL and treatment burden of patients with stage III-N2 and their carers. The breadth of data showed effective trial processes such as good trial explanation, clinicians’ active MDT attendance and addressing patients’ preconceptions. Patients and carers spoke about the experience of taking part in PIONEER, emphasising the demanding treatment schedules of chemotherapy and radiotherapy, the intense recovery from surgery, the psychological impact in both arms, maintaining a positive mindset during this tumultuous time, and despite feeling well supported, often needing further treatment information. Interviews with patients who declined participation highlighted that treatment experience and preference, concerns and not understanding randomisation, all influenced their decision. These patients often wanted to make their own treatment decision with support from family and friends. Similarly, Harrop *et al* found that decliner patients preferred one surgery type over another and often had an unwillingness to surrender control to randomisation.^24^ During interviews with consented patients, we found that once the trial had been explained in detail, they felt more comfortable with the randomisation process.

Consistent with findings from other areas and earlier-staged lung cancer, the main barriers to recruitment and decision-making within MDTs were clinicians and system-related issues, where they often held preconceived views on the most appropriate treatment and demonstrated bias towards their own specialty.^15 25^ Clinicians reported that recruiting to PIONEER was more time-consuming and complex than competing trials and noted that having a dedicated PIONEER champion, someone who worked closely with the research team and was solely focused on recruiting for PIONEER, attending MDTs would have been beneficial. This was unlikely to be feasible in reality given the number of sites included and the limited resources.

In both interviews and diaries, patients and carers reported disruption to daily life due to frequent appointments on the non-surgical arm, the challenge of adapting to a ‘new normal’, and the burden of caring responsibilities. Patients often reported frustration linked to loss of independence, concerns about being a burden, and fluctuating moods, leading to increased reliance on others. Adam *et al* described cancer as a threat to personal identity through these profound lifestyle changes and loss of independence.^26^ Although patients expressed anxiety and low mood, carers reported experiencing similar emotions, though often concealed to avoid further upset to the patient. Evidence suggests that the HRQoL of cancer carers is disproportionate when compared with carers of people with other chronic diseases.^27^ Patients voiced deep appreciation for carer support, while carers valued having access to a broader support network. Support and clear communication to both patients and carers were viewed as invaluable throughout the trial; however, additional support was particularly needed before surgery, and consistent communication should be maintained post-treatment.

Patients in both trial arms experienced differing and complex side effects and treatment burdens, as demonstrated by the quantitative and qualitative findings of the patient and carer diaries. Those randomised to the non-surgical arm were most negatively affected in the first 12 weeks, largely due to the daily demands of radiotherapy appointments, whereas surgical patients faced the greatest challenges in the first 6 weeks. While patients’ mood fluctuated over time, carers reported greater distress during the early weeks of treatment, reflecting worries about the course of treatment and the weight of caring responsibilities. Providing tailored information about the different treatment arms and when patients and carers are most likely to experience these burdens may help to better prepare and support them.

When asked about considerations for future trials, patients described the number and repetitiveness of the questionnaires as burdensome and several would have preferred an online format. Clinicians shared concerns about questionnaire burden, suggesting it may have discouraged some patients from consenting. It is important to note that trial evidence does not support a change of mode alone as a solution: an updated Cochrane review found response rates were higher with postal than electronic questionnaires, and offering a choice of mode conferred no advantage over postal alone.^28^ Our own data are consistent with this, in that the documented reasons for non-completion were predominantly illness and hospitalisation rather than difficulties with the format. Taken together, this suggests that improving completion in this population will depend less on the mode of administration than on reducing the number of instruments, minimising repetition, and offering supported completion in clinic or by telephone, with a choice of format retained on grounds of patient preference and acceptability. As supported by previous research,^25^ to ensure treatments are presented with equipoise, MDT members recommended that trial information should be delivered by a representative from each profession (eg, surgeon, respiratory physician, research nurse). Given the rapidly evolving treatment landscape for this cohort of patients, clinicians also highlighted the importance of a flexible protocol that could be adapted to incorporate new options as they emerge.

The qualitative findings help to explain the recruitment difficulties observed in PIONEER and should be interpreted alongside the quantitative results reported in the main study.^17^ Of all patients screened, 81% were deemed ineligible for randomisation, primarily due to physiological fitness (47%), resectability concerns (30%) or both factors (16%). Furthermore, central review identified that 8 out of 65 (12%) cases initially classified as unresectable were potentially eligible, highlighting the degree of variation in resectability assessment across centres. These findings were strongly reflected in clinician interviews, with participants describing how treatment recommendations and judgements of resectability could vary depending on the surgeons involved in multidisciplinary discussions. Similarly, concerns regarding resectability are consistent with previous work from the European Organisation for Research and Treatment of Cancer (EORTC) group,^29–31^ reinforcing the persistent challenges associated with defining and managing resectable stage III disease. Clinicians also emphasised the limited pool of eligible patients and the challenges of conducting a trial within a rapidly evolving therapeutic landscape, while noting that the overwhelming majority of patients would ultimately not enter the study because of ineligibility or treatment preferences. Taken together, these qualitative and quantitative findings suggest that the recruitment challenges encountered by PIONEER were largely driven by structural features of the stage III-N2 NSCLC population and clinical decision-making processes, rather than shortcomings in trial conduct. This may explain why similar accrual difficulties have been observed across previous multimodality studies, including EORTC 08941, Intergroup-0139, ESPATUE and SAKK.^5–8^ Consequently, future research may benefit from alternative approaches, such as real-world observational designs and registry-based studies, while also focusing greater attention on the substantial proportion of patients who remain ineligible for current treatment pathways.

### Strengths and limitations

A strength of this study is that the findings are derived following the triangulation of data collected from four different sources and from three different participant groups, providing a rich insight into experiences. The inclusion of participants who declined to take part provides valuable information for future research in this area. A further strength is that MDT interview participants were purposively sampled according to the range of eligible patients at their site, with no themes more prevalent in any eligibility group. Sampling in this way ensured we collected the full range of experiences of recruiting to PIONEER. Despite the strengths highlighted, there are a number of limitations. We aimed to involve nursing staff in MDT interviews, as they can provide valuable insights into patient care and functioning; however, no research nurses volunteered. As a result, the findings predominantly reflect a medical perspective. While these yielded sufficient data to explore key themes, there is a risk that these professionals’ views were biased, limiting the generalisability and conclusions that can be drawn from these interviews. The sample size for patients consented to the main study is small; we do however have a wealth of data from a variety of different sources collected from this small patient group and MDT members. We did not have representation from all sites in the recruitment consultations and interviews meaning findings may not be representative.

## Conclusions

Through the rich data generated from multiple sources, this study provides valuable insights into the experiences of patients, carers, and clinicians participating in the PIONEER trial. The findings highlight important practical and methodological challenges that would need to be addressed in any future large-scale multisite study. However, in the context of an evolving treatment landscape, the priority may no longer be to determine which treatment strategy is superior, but rather to understand why a substantial proportion of patients remain ineligible for potentially beneficial therapies and how access to optimal treatment can be improved. Notably, the recruitment and eligibility challenges observed in PIONEER are consistent with those reported in previous multimodality trials, reinforcing the longstanding difficulties associated with conducting research in this patient population. Future research should therefore consider alternative approaches, including the use of real-world data alongside or in place of traditional randomised trial designs, reduced and less repetitive outcome measures with supported and flexible completion, and adaptive study protocols that can respond to rapidly evolving standards of care. Ultimately, improving equitable access to effective treatments for all eligible patients may represent a more clinically meaningful objective than further comparisons between competing treatment modalities.

## Supplementary material

10.1136/bmjresp-2026-004632 online supplemental file 1

10.1136/bmjresp-2026-004632 online supplemental file 2

10.1136/bmjresp-2026-004632 online supplemental file 3

## Data Availability

All data relevant to the study are included in the article or uploaded as supplementary information.
