# Supplementary material for: Experience of participating in a randomised controlled feasibility trial of surgery versus no surgery as part of multimodality treatment in stage III-N2 non-small cell lung cancer: PIONEER
Source: BMJ Open Respir Res. 2026 Oct 26;13(1):e004632. doi: 10.1136/bmjresp-2026-004632 (PMC13711569; doi:10.1136/bmjresp-2026-004632)
Supplement: Supplementary file 2 [file bmjresp-13-1-s002.docx]

## Supplemental Table 2: Participant characteristics

| **Consented Patients** N=28 | **Consultation recordings** | **Diaries** | | **Interviews** |
| --- | --- | --- | --- | --- |
| **Total** n (%) | 12 (42.9) | 21 (75) | | 12 (42.9) |
| **Gender** n (%)  Male  Female | 4 (33.3)  8 (66.7) | 11 (52.4)  10 (47.6) | | 7 (58.3)  5 (41.7) |
| **Age** mean | 67 | 66 | | 66 |
| **Ethnicity** n (%)  White British  Irish  Caribbean  African  Any other ethnic group  Missing | 10 (83.4)  0 (0)  0 (0)  1 (8.3)  0 (0)  1 (8.3) | 15 (71.4)  1 (4.8)  1 (4.8) 0 (0)  2 (9.5)  2 (9.5) | | 10 (83.4)  1 (8.3)  0 (0)  0 (0)  1 (8.3)  0 (0) |
| **Performance status**** n (%)  0  1  2 | 2 (16.7)  9 (75)  1 (8.3) | 5 (23.8)  15 (71.4)  1 (4.8) | | 4 (33.3)  7 (58.4)  1 (8.3) |
| **Carers** N=9 | **Diaries** | | **Interviews** | |
| **Total** n (%) | 8 (88.9) | | 2 (22.2) | |
| **Gender** n (%)  Male  Female | 3 (37.5)  5 (62.5) | | 1 (50)  1 (50) | |
| **Age** mean | 51* | | 34* | |
| **Relationship to patient** n (%)  Relative  Friend  Other  Missing | 5 (62.5)  1 (12.5)  1 (12.5) 1 (12.5) | | 1 (50)  1 (50)  0 (0) | |
| **Ethnicity** n (%)  Welsh/English/Scottish/Northern Irish/British | 8 (100) | | 2 (100) | |
| **Employment status** n (%)  Employed  Self-employed  Retired from paid work  Unable to work because of long-term disability or ill-health  Looking after the family or home  Missing | 2 (25)  1 (12.5)  2 (25)  1 (12.5)  1 (12.5)  1 (12.5) | | 2 (100)  0 (0)  0 (0)  0 (0)  0 (0)  0 (0) | |
| **Clinicians** N=20 | **Interviews** | | | |
| **Profession** n (%)  Oncologist  Surgeon  Respiratory Physician | 4 (20)  6 (30)  10 (50) | | | |
| **Key:** *= age missing for one carer; ** European Cooperative Oncology Group Performance Status which is widely used to standardise assessment of a patients’ function. Scores range from 0 to 4 with 0 indicating fully active and 4 completely disabled[24]. | | | | |
