# Supplementary material for: Experience of participating in a randomised controlled feasibility trial of surgery versus no surgery as part of multimodality treatment in stage III-N2 non-small cell lung cancer: PIONEER
Source: BMJ Open Respir Res. 2026 Oct 26;13(1):e004632. doi: 10.1136/bmjresp-2026-004632 (PMC13711569; doi:10.1136/bmjresp-2026-004632)
Supplement: Supplementary file 3 [file bmjresp-13-1-s003.docx]

## Table 3: Participant quotes. Patient and carer sex and age are provided and clinicians’ professional group and their sampling group based on the level of patient eligibility at their site.

| **Theme 1: Trial processes** | |
| --- | --- |
| Interviews with declining patients | *“I had already had surgery before, I had half my right lung taken away, three years ago, and I didn’t want opened up again…” (Declining Patient 07, No demographics collected).*  *“…I wanted to get it over and done with for psychological reasons. So I know surgery is going to come with complications but I’d rather just know that it’s out of my body…” (Declining Patient 403, No demographics collected).*  *“Oh there was only one reason, I wanted to take control, have control over what treatment I was going to receive. I was happy with everything except the fact that the computer would decide.” (Declining Patient 403, No demographics collected).* |
| Interviews with consenting patients | *“He explained then about whether there would be surgery or the treatments of chemo or radiotherapy so he went through all of that very clearly…” (Patient 111, Male, 70).*  *“…I understand randomisation…so I was more than happy to be entered into that lottery for research purposes.” (Patient 404, Male, 57)*  *“I just thought if I could help someone else in any way I could, that’s why I did it love…” (Patient 206, Female, 51).* |
| MDT interviews: Clinician role | *“…I think that’s probably the single biggest facilitator…having a champion of the study.” (Clinician 1, Oncologist, low ineligibility).*  *“I think having a trial that compares such different treatments having an operation vs radiotherapy was always going to be challenging and we knew that from the beginning.” (Clinician 22, Respiratory Physician, no eligible patients).* |
| MDT interviews: Patient factors | *“…if you ask a hundred patients who can have cancer removed…to be randomised in a trial with questionnaire outcomes I think 95 out of 100 will say no.” (Clinician 2, Surgeon, low ineligibility).*  *“…it’s just a difficult field isn’t it because the numbers are small and as I’ve said already the treatment paradigm is changing rapidly…to get sufficient numbers for a full trial is going to be difficult.” (Clinician 3, Respiratory Physician, average ineligibility).* |
| MDT interviews: System issues | *“PIONEER is not the only study…it’s a busy MDT with a lot of research commitment…” (Clinician 24, Surgeon, high ineligibility).*  *“So I think it's just the support that we currently have from our trial unit that lost a lot of staffing, and they’re really thin on the ground” (Clinician 23, Respiratory Physician, average ineligibility).* |
| MDT interviews: Decision-making | *“…they had N2 disease, but they may not have been surgically resectable, but that opinion varied depending on which surgeon you had in the MDT.” (Clinician 9, Respiratory Physician, no eligible patients).*  *“…patients don’t, generally don’t like to make their own decisions even though we obviously want, want to make sure every decision is a shared decision.” (Clinician 6, Respiratory Physician, average ineligibility).* |
| **Theme 2: Experience of taking part in PIONEER** | |
| Physical impact | *“… All my hair fell out. And it was tiredness, nausea, it was all the usual things. I lost, you know, taste, everything tasted like metal, and really tired all the time.” (Patient 104, Female, 63).*  *“…I feel that with regard to chemo I've been very well supported from a medical point of view.” (Patient 121, Male, 67).*  *“I’m stressed from the lack of sleep.” (Carer 1011, Female, 62).* |
| Impact on daily lives | *“I use the hospital transport because my husband doesn’t drive…I was being ready at 9 o’clock in the morning…and not getting back till about somewhere between 12 and 1.” (Patient 106, Female, 82).*  *“Well, when I was going through the six weeks, all I was doing was being at the hospital all day every day…” (Patient 104, Female, 63).*    *“Work’s been good, giving me the time off, but I’m having to pay back all the hours.” (Carer 2061, Male, No age provided)* |
| Psychological impact | *“…We both had a serious meltdown right at the beginning of it all…” (Patient 121, Male, 67).*  *“…I got odd days I got very down but it was only, I think again because I was tired by the evening, I thought I can’t cope anymore with this damn thig, I got very low…” (Patient 106, Female, 82).*  *“…There’s lots of decisions that need to be made that could be life altering, so yeah, psychologically I think it’s a tough period for anybody to go through and sort of sit on the sidelines and be helpless…that feeling of being out of control was constant” (Carer 1211, Female, 34).*  *“I feel my mood has been better this week; due to the results of the ultrasound it had proved not to be anything sinister.” (Carer 1011, Female, 62)* |
| Coping mechanisms | *“My husbands been a god send, he’s helped me with the housework and if I’m too tired at tea…he’s been my rock.” (Patient 206, Female, 51).*  *“I wouldn’t say it was a burden because you know I’d do anything for a friend or family, or somebody I was caring for…” (Carer 1211, Female, 34).*  *“…I could contact either the oncology nurse or the MacMillan support team…I knew they would return my calls if I needed any support.” (Patient 404, Male, 57).* |
| **Theme 3: Considerations for a future trial** | |
| Interviews with consenting patients and their carers | *“…it would have been a lot easier to have done it online rather than filling the paperwork in, then folding it up and then waiting for the stamped addressed envelopes to turn up and then putting it in the envelopes and posting...” (Patient 121, Male, 67).*  *“…I struggled to keep on top of them [questionnaires] so yeah I think I understand like they were important to do but I think trying to balance my already hectic work schedule and hectic personal life with caring for somebody and the sort of impact that sort of has on my personal life…” (Carer 1211, Female, 34).* |
| Interviews with MDT members | *"...the thing that we really need to nail down is with the current treatment options which group does genuinely have clinical equipoise..." (Clinician 3, Respiratory Physician, average ineligibility).*    *"...that patient that was happy to enter the study but not complete the questionnaires, if there was an option to I don't know, reduce the set or something that might have helped." (Clinician 1, Oncologist, low ineligibility).*    *"I just wonder if it [trial information] could have been better disseminated, I don't think I was aware of PIONEER until someone pushed that through a third party." (Clinician 11, Respiratory Physician, no eligible patients).* |
